# Immunogenicity of a Trivalent Recombinant Vaccine Against *Clostridium perfringens* Alpha, Beta, and Epsilon Toxins in Farm Ruminants

**DOI:** 10.1038/srep22816

**Published:** 2016-03-23

**Authors:** Gustavo Marçal Schmidt Garcia Moreira, Felipe Masiero Salvarani, Carlos Eduardo Pouey da Cunha, Marcelo Mendonça, Ângela Nunes Moreira, Luciana Aramuni Gonçalves, Prhiscylla Sadanã Pires, Francisco Carlos Faria Lobato, Fabricio Rochedo Conceição

**Affiliations:** 1 Centro de Desenvolvimento Tecnológico, Universidade Federal de Pelotas, Pelotas, Rio Grande do Sul, CEP 96160-000, Brazil; 2 Instituto de Medicina Veterinária, Universidade Federal do Pará, Castanhal, Pará, CEP 68740-970, Brazil; 3 Faculdade de Nutrição, Universidade Federal de Pelotas, Pelotas, Rio Grande do Sul, CEP 96010-610, Brazil; 4 Escola de Veterinária, Universidade Federal de Minas Gerais, Belo Horizonte, Minas Gerais, CEP 30123-970, Brazil

## Abstract

*Clostridium perfringens* is an anaerobic bacterium that produces several toxins. Of these, the alpha, beta, and epsilon toxins are responsible for causing the most severe *C. perfringens*-related diseases in farm animals. The best way to control these diseases is through vaccination. However, commercially available vaccines are based on inactivated toxins and have many production drawbacks, which can be overcome through the use of recombinant antigens. In this study, we produced recombinant alpha, beta, and epsilon toxins in *Escherichia coli* to formulate a trivalent vaccine. Its effectiveness was evaluated through a potency test in rabbits, in which the vaccine generated 9.6, 24.4, and 25.0 IU/mL of neutralizing antibodies against the respective toxins. Following this, cattle, sheep, and goats received the same formulation, generating, respectively, 5.19 ± 0.48, 4.34 ± 0.43, and 4.70 ± 0.58 IU/mL against alpha toxin, 13.71 ± 1.17 IU/mL (for all three species) against beta toxin, and 12.74 ± 1.70, 7.66 ± 1.69, and 8.91 ± 2.14 IU/mL against epsilon toxin. These levels were above the minimum recommended by international protocols. As such, our vaccine was effective in generating protective antibodies and, thus, may represent an interesting alternative for the prevention of *C. perfringens*-related intoxications in farm animals.

*Clostridium perfringens* is a ubiquitous anaerobe bacillus that can be found in soil and water, as well as in the microbiota of many animals. This spore-forming bacterium produces 16 different toxins, but only four of them—alpha, beta, epsilon, and iota—are considered the most important since they are related to the pathogenesis of most of the *C. perfringens*-related diseases. Based on the production profile of these four toxins, *C. perfringens* is classified into five types, from A to E. Each of these types are related to the development of certain diseases in different animal species, since different species exhibit distinct susceptibility to these toxins^1^.

The most important *C. perfringens* types in agricultural veterinary medicine are C and D, since they affect the majority of farm animals. The former produces alpha and beta toxins, and can cause several diseases, mainly necrotizing enteritis, in many farm animals such as cattle, sheep, goats, and swine. The latter produces alpha and epsilon toxins and causes enterotoxemia in cattle, sheep, and goats. *Clostridium perfringens* type B, which produces the three toxins, is also important since it causes diseases similar to type C^2^. Usually, these diseases are highly debilitating and lead to sudden death. Other *C. perfringens* types (A and E) are also associated with intestinal diseases, both in farm animals and humans, but to a lesser extent. Although the role of alpha toxin in the pathogenesis of intestinal diseases in mammals is still not fully understood, it is considered the main causative agent of gas gangrene, which is mainly related to *C. perfringens* type A infections^3^. This disease results from the pathogen coming into direct contact with damaged skin or muscle tissues, and can affect both animals and humans^1^,^4^. On the other hand, the effect of both beta and epsilon toxins is very well defined, and they are commonly understood to be the main factors responsible for the diseases caused by *C. perfringens* types C and D, respectively. As such, alpha, beta, and epsilon toxins are the major toxins involved in *C. perfringens* pathogenesis among several animal species, including cattle, sheep, and goats, and, thus, are the focus of the majority of existing studies in this field.

In Brazil alone, there are more than 200 million bovines, 18 million sheep, and 14 million goats. The country has the 2^nd^ largest cattle herd in the world and is the world’s largest cattle meat exporter, selling to more than 180 countries. Furthermore, it is the 18^th^ largest country in the world in terms of the exportation of caprine products^5^. Since these three farm animals are susceptible to *C. perfringens* toxins and the eradication of the diseases caused by these toxins is almost impossible, vaccination represents the best approach through which to control these diseases. The commercial vaccines that are currently available are polyvalent and based on toxoids (inactivated toxins). However, the process by which these toxoids are produced has some drawbacks since it requires complex components in culture medium and is potentially dangerous because *C. perfringens* is pathogenic for humans. The high variability between different fermentation batches requires continuous selection of strains that exhibit satisfactory toxin production. This increases the complexity of the process and does not result in an effective increase in yield^5^. Furthermore, the inactivation step, which involves the use of formaldehyde, is very time-consuming, taking about ten days^6^.

It is possible to reduce biosafety problems by using *Escherichia coli* BL21 (DE3) as an expression system to obtain recombinant vaccine antigens, since this strain is not pathogenic. Furthermore, protein yield variations would be solved once conditions for production in this expression system are very well-defined^7^. The overall production time can also be reduced because *E. coli* can yield large amounts of antigen that are usually less toxic or non-toxic. As such, the present study aimed to develop a trivalent recombinant vaccine against the three major *C. perfringens* toxins—alpha, beta, and epsilon—and to evaluate the efficacy of this vaccine in cattle, sheep, and goats.

## Results

### Production of recombinant toxins

**Figure 1:**
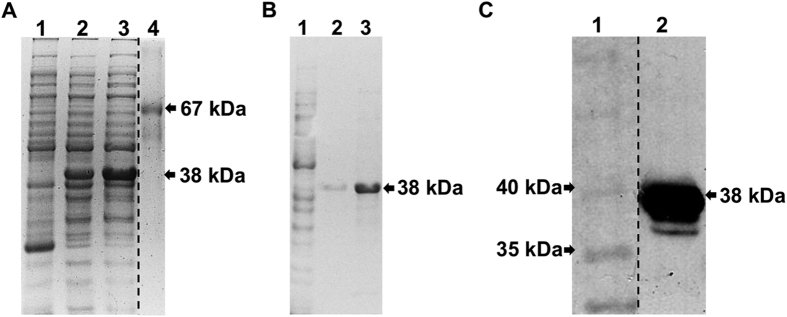
SDS-PAGE 12% and Western blot using anti-6xHis antibody for the detection of rEpsilon. (**A**) Solubilization of rEpsilon (38 kDa) expressed in *E. coli* BL21 (DE3) Star. 1- *E. coli* BL21 (DE3) Star not transformed; 2- induced *E. coli* BL21 (DE3) Star transformed with pAE-rEpsilon; 3- supernatant of lysis buffer of induced *E. coli* BL21 (DE3) Star containing pAE-rEpsilon; 4–BSA (67 kDa). (**B**) Elution fractions of rEpsilon after Ni^ + 2^-affinity purification. 1- supernatant of lysis buffer of induced *E. coli* BL21 (DE3) Star containing pAE-rEpsilon after purification; 2-3- purified rEpsilon. (**C**) Western blot using anti-6xHis against purified rEpsilon. 1- PageRuler Prestained Protein Ladder (Thermo Scientific); 2- purified rEpsilon. Of note, (**A,C**) parts of this figure were cropped from a single image on the dashed lines to be better presented in the article’s context, although the gels have been run under the same conditions and the Western blot performed with the same set of materials. These two complete figures can be found, respectively, as Supplementary Figs S1 and S2.

The expression of rAlpha and rBeta exhibited the same pattern as that previously described^8^,^9^. The former was expressed and purified from the soluble fraction, from which it could be detected by Western blot using anti-6xHis monoclonal antibody after purification. The latter was present in the insoluble fraction, where it was detected, solubilized using urea 8 M, and purified. Similar to rAlpha, rEpsilon was present in the soluble fraction, detected by Western blot and purified (Fig. 1). The three proteins exhibited the expected molecular weight, which were 48, 37, and 38 kDa for rAlpha, rBeta, and rEpsilon, respectively.

### Vaccine formulation is sterile and recombinant epsilon antigen is non-toxic

The recombinant vaccine formulation used in this work contained 200 μg of each recombinant antigen and Al(OH)_3_ as adjuvant. The sterility test resulted in no growth under both aerobic and anaerobic conditions, indicating the formulation was not contaminated. Since our group has previously demonstrated that rAlpha and rBeta are non-toxic^9^, only rEpsilon was tested for toxicity in this study. The MTT (3-[4,5-dimethylthiazol-2-yl]-2,5-diphenyltetrazolium bromide) cytotoxicity assay using MDCK (Madin-Darby canine kidney, ATCC) cells treated with the recombinant antigen exhibited no difference in absorbance when compared to the negative control group for any of the concentrations tested (data not shown). This result indicates that, similarly to rAlpha and rBeta, rEpsilon is non-toxic and does not need to be inactivated.

### The trivalent vaccine generates antibody levels higher than the minimum required

New Zealand rabbits were immunized with the formulation described in the previous paragraph via a two-dose scheme, and the production of neutralizing antibodies was measured by seroneutralization in mice. The results of the potency test are shown in Table 1. The antitoxin levels considered for this test are based on the rules described by international protocols that are also specified in Brazilian legislation, which recommends a minimum of 4, 10 and 2 IU/mL against alpha, beta, and epsilon toxins, respectively^10^,^11^,^12^.

Our formulation exceeded all of these minimum antitoxin levels, since neutralizing antibody levels against alpha, beta, and epsilon were 5.6, 14.4, and 23 IU/mL respectively and, therefore, higher than the recommended levels of antibodies for commercial vaccines in Brazil. Furthermore, the recombinant vaccine exhibited antibody levels of 2.7, 2, and 13 IU/mL higher than the commercial vaccine against the respective toxins. The group that received the PBS (negative control) had no detectable antibody levels against either toxin and, therefore, the data pertaining to this group is not included in Table 1.

### Farm animals produce protective humoral immunity when immunized with the trivalent vaccine

In order to verify the results obtained when using rabbits as the animal model, additional target species animals were also immunized to evaluate the trivalent vaccine. The species employed were cattle, sheep, and goats. Three groups were designed to evaluate the vaccine for each species, including a group that was vaccinated with the recombinant formulation, one with a commercial vaccine, and another with vehicle (only aluminum hydroxide in PBS, negative control). Unlike the potency test, sera were not pooled, but individually analyzed. Tables 2, 3 and 4 show the results obtained for each serum sample after the seroneutralization was performed in mice. In cattle, the established minimum antitoxin levels were surpassed by 1.19, 3.71, and 10.74 IU/mL for alpha, beta, and epsilon toxins, respectively. In sheep, the antibody levels were also higher than the minimum antibody levels, this time by 0.34, 3.71, and 5.66 IU/mL respectively; and, in goats, these numbers were 0.70, 3.71, and 6.91 IU/mL respectively.

**Figure 2:**
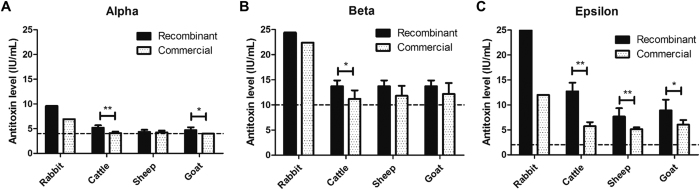
Antitoxin level generated by model (rabbit) and ruminant (cattle, sheep, and goat) species. All groups matched or surpassed the minimum level required (dashed line), which are 4, 10, and 2 IU/mL against alpha, beta, and epsilon toxins, respectively. (**A**) Level of neutralizing antibodies against alpha toxin generated by each of the species. When recombinant and commercial vaccines are compared among the ruminants, cattle and goat showed significant higher levels, but not for sheep. (**B**) Level of neutralizing antibodies against beta toxin generated by each of the species. Only cattle showed significant higher levels when comparing recombinant and commercial vaccines in farm animals. (**C**) Level of neutralizing antibodies against epsilon toxin generated by each of the species. By comparing recombinant and commercial vaccines, the former one generated higher antitoxin levels for every ruminant species. *P < 0.05; **P < 0.01.

When the antibody levels of the recombinant vaccine were compared with those of the commercial vaccine, the former exhibited higher mean levels of neutralizing antibody. In cattle, the recombinant formulation was capable of inducing levels of 1.08, 2.51, and 7 IU/mL higher than the commercial vaccine against alpha, beta, and epsilon toxins, respectively. In sheep, these values were 0.11, 1.88, and 2.52 IU/mL higher, while, in goats, they were 0.7, 1.54, and 2.85 IU/mL higher than the commercial vaccine. According to the statistical analysis, rAlpha was able to induce significantly higher responses in cattle (P < 0.01) and goats (P < 0.05), but not in sheep (Fig. 2A). rBeta antigen induced significantly higher levels only in cattle (P < 0.05; Fig. 2B), while rEpsilon was able to induce significantly higher levels in all three species (P < 0.01 for cattle and sheep, and P < 0.05 for goats; Fig. 2C). The negative controls of each species did not contain any detectable neutralizing antibody levels and were, therefore, omitted from Tables 2, 3 and 4.

### Neutralizing antibody levels induced in ruminants are lower than in rabbits

Following the administration of the recombinant vaccine, the antibody levels induced in rabbits, which is the animal model for testing *C. perfringens* vaccines, were considerably higher than those obtained in cattle, sheep, and goats. Statistical analysis indicated that the three groups of farm animals immunized with the recombinant formulation had significantly lower immune responses than rabbits (P < 0.05). Approximately 2- to 3-fold lower immune response occurred in farm species in comparison to rabbit antibody levels. The difference between rabbits and ruminants ranged from 4.41 to 5.26 IU/mL for the alpha toxin; while this difference was 8.69 and ranged from 12.26 to 17.34 IU/mL for beta and epsilon, respectively. A similar disparity was also observed for the commercial vaccine (P < 0.05), with the difference ranging from 5.37–5.60, 10.23–11.20, and 18.94–19.86 IU/mL against the respective toxins.

## Discussion

The production of vaccines that can protect against *Clostridium* spp. has been continually reported as problematic due to factors related to the culture of these pathogenic microorganisms and how they behave under anaerobic conditions^13^,^14^,^15^. The process used in this work involved simpler procedures and materials than those used to develop commercial vaccines, and, therefore, has the potential to overcome most of the problems related to the industrial scale production of such vaccines. Furthermore, the production and use of the recombinant antigens described herein are safer, since the *E. coli* BL21 (DE3) strain is not pathogenic and the resulting antigens exhibited no signs of toxicity or hypersensitivity in any of the animals tested. The fact that these recombinant toxins do not need to be inactivated after purification is also an advantage, since this step is time-consuming and confers a risk of hypersensitivity in the vaccinated individual due to residual formaldehyde^6^.

In Brazil, more than 200 million animals have to be vaccinated on an annual basis, requiring a large amount of doses for this expanding market^16^. Vaccines against *C. perfringens* are very important for animal breeding and, thus, developing a novel production process for *C. perfringens* vaccines could reduce the cost of the products used to vaccinate agricultural animals. The huge impact of *C. perfringens*-related intoxications on the market has motivated studies all over the world, and many researchers have attempted to develop new vaccines, especially those that use recombinant DNA technologies, which can overcome the problems associated with conventional vaccines^15^,^17^,^18^.

Some works have reported the use of recombinant *C. perfringens* toxins as vaccine components. Our group recently tested rBeta in rabbits, which induced 14 IU/mL of neutralizing antibodies with 100 μg per dose^8^. Another study published by our group involved the concomitant use of rAlpha and rBeta in swine^9^. In this case, pregnant sows that were immunized with 200 μg per dose of each antigen exhibited adequate levels of neutralizing antibodies (6.0 and 14.5 IU/mL against alpha and beta, respectively). In the same study, piglets were also protected by passive immunization after colostrum intake and exhibited 4.2 and 9.6 IU/mL against the respective toxins. The use of a fusion recombinant antigen containing alpha, beta, and beta 2 protein sequences was also efficient in eliciting a protective immune response in pregnant sows and cows^19^. In this study, 100 μg per dose were able to induce neutralizing antibodies against a culture filtrate of *C. perfringens* type C, although it was not clear if this immune response was at the minimum levels of antibodies necessary to protect farm animals. Another fusion strategy, this time with beta and epsilon sequences, was able to elicit 10 and 6 IU/mL of neutralizing antibodies against the respective toxins in rabbits^17^. In another work, immunization with 200 μg of insoluble rEpsilon alone induced 13.1, 26.0, and 14.3 IU/mL of protective antibodies in cattle, sheep, and goats, respectively^20^.

Considering that all three of these recombinant toxins have been proven to elicit neutralizing antibodies against the native toxins, a recent work tested a trivalent vaccine in cattle^18^. In this study, the recombinant C-terminal region of alpha, and complete versions of beta and epsilon toxins, all expressed separately, were used to develop vaccines that contained 200 μg of each antigen per dose together with an oil adjuvant. When injected separately, cattle generated 23.04, 33.70, and 9.43 IU/mL against the respective toxins. However, the results were converted from non-standard tests, rather than calculated directly in IU/mL, and, as commented by the authors, these conversions may have reduced the precision of the results. Moreover, antibody levels of the group immunized with the three antigens together were not mentioned, although it was reported that this group exhibited similar antibody levels against each of the antigens. In the present study, 200 μg of each of the three antigens were used to formulate the recombinant trivalent vaccine using Al(OH)_3_ as adjuvant. In this case, the vaccine was able to induce 5.19, 13.71, and 12.74 IU/mL in cattle. These titers were lower than those described by Jiang *et al*.^18^, but offered the advantage of using the well-known and low-cost Al(OH)_3_ rather than an oil adjuvant. Nevertheless, the vaccine-induced neutralizing antibody levels were still above the minimum titers recommended by international directives from the USDA^25^, European Pharmacopoeia^26^, and CFR9^27^ that are also used in Brazil, which are 4, 10 and 2 IU/mL against alpha, beta, and epsilon toxins, respectively. It is worth to notice that both studies showed only the potential of the recombinant vaccines in eliciting neutralizing antibodies through seroneutralization assay in mice. This technique alone cannot conclusively indicate protection in farm animals, although it shows that the described formulations have potential to be used as effective vaccines. Thus, further investigation in field experiments are required to completely define their effectiveness.

Since conventional vaccines do not rely on direct quantification of antigens, but on their ability to generate protective antibodies, a large batch-to-batch variation in the number of doses produced is likely to occur^3^,^13^. Thus, the use of recombinant antigens offers the possibility of overcoming this problem, once they can be easily quantified and related to the protective levels generated in animals. Considering only the values obtained in rabbits in the present study, lower quantities of the antigens (<200 μg), especially rBeta and rEpsilon, could also provide protective immunity in the potency test without compromising vaccine efficacy. Similarly, the tests in farm animals identified higher levels of anti-toxins against beta and epsilon, indicating that diminishing the quantity of rBeta and rEpsilon will not compromise its efficacy. However, when comparing recombinant and commercial vaccines tested in farm animals, it became clear that only rEpsilon could be reduced in the formulation to develop a recombinant vaccine similar to the commercial offering. Nevertheless, the commercial vaccine used in this study was formulated to protect against nine different pathogens from the *Clostridium* genus, while our formulation can protect against only four. Although further tests in combination with other antigens are still required to develop a comprehensive comparison of the vaccines, 200 μg is, in principle, the appropriate amount of rAlpha and rBeta, while lower rEpsilon amounts should be considered. On the other hand, the use of larger amounts of antigen in each dose can increase the longevity of the immune response and, thus, reduce the frequency of injections required to maintain immunoprotection.

There was also a discrepancy in the results between the antibody levels in rabbits and those in farm animals measured by seroneutralization in mice after immunization. This model-to-target reduction in neutralizing antibody levels was also observed in previous studies that used *Clostridium botulinum* vaccines composed of recombinant H_C_C and H_C_D antigens^21^,^22^. The animal model in these previous studies was Guinea pig and, although the antibody level against botulinum toxin type C was the same when compared to cattle (5.0 IU/mL), the antibody level against botulinum toxin type D was lower in cattle (6.14 IU/mL, compared to 10.0 IU/mL in Guinea pigs). Another study published by our group, which involved a bivalent vaccine that contained rAlpha and rBeta, also exhibited this same variation^9^. Neutralizing antibody levels in rabbits were 9.6 and 20.4 IU/mL against the respective toxins, while in swine they were 6.0 and 14.5 IU/mL, respectively. This immune response behavior was also observable in studies with rEpsilon alone^20^,^23^. In this case, neutralizing antibody level in rabbits was 40.0 IU/mL; while, in cattle, sheep, and goats, it was 13.1, 26.0, and 14.3 IU/mL, respectively. These last results corroborate with those presented in this paper, since a reduction in immune responses of approximately two- to three-fold was observed in ruminants. Although the frequency of this reduction in every farm species is still very much subject to speculation, it should be taken into account when performing vaccine studies with target species, since it can be helpful when designing i.e. longevity experiments. It may also be important to highlight the fact that neutralizing antibody levels against epsilon toxin achieved superior results (approximately two-fold higher) for the recombinant vaccine than the commercial vaccine in cattle, suggesting that our rEpsilon stimulates a higher immunological response in this species.

The vaccine described here showed to be effective in eliciting levels of neutralizing antibodies higher than required by international standards, as accessed by seroneutralization in mice. Thus, it may represent an effective alternative for the prevention of *C. perfringens*-related diseases in farm animals, although further studies to prove its efficacy in the field are still needed. As previously reported, *C. perfringens* recombinant toxins were able to elicit protective antibodies in swine and cattle^9^,^18^,^19^. Now, sheep and goats are also known to generate the same kind of response when receiving recombinant toxin vaccines. Additional studies can be performed to adapt the production of the antigens to an industrial scale, using fermenters, as well as to evaluate the longevity of the immune response in combination with other antigens.

## Methods

### Expression and purification of recombinant alpha, beta, and epsilon toxins

The production of recombinant alpha (rAlpha) and beta (rBeta) toxins was conducted as previously described using genes obtained by PCR from *C. perfringens* type C genomic DNA^8^,^9^. The gene for recombinant epsilon (rEpsilon) was synthesized (Epoch Life Sciences) containing preferential codons for *E. coli* expression based on the amino acid sequence from GenBank (gi: 209947607) but without the 45 N-terminal amino acids. The gene was then subcloned into pAE expression vector^24^ following a previously described methodology^25^. rEpsilon was produced in *E. coli* BL21 (DE3) Star, which was cultured in Luria-Bertani (LB) medium with ampicillin 100 μg/mL. A pre-inoculum with 50 mL of this medium was cultured for 16 h at 37 °C and 200 RPM and then transferred to 450 mL. When the 600 nm-optical density reached 0.5–0.8, protein expression was induced by adding IPTG (isopropyl β-D-1-thiogalactopyranoside) to a final concentration of 0.5 mM, and the culture was kept under the same conditions for 4 h. Cells were harvested by centrifugation (10,000 × g; 10 min; 4 °C), suspended in lysis buffer (NaH_2_PO_4_ 0.2 M; NaCl 0.5 M; Imidazole 10 mM; lysozyme 100 μg/mL), sonicated three times for 20 s and centrifuged again. Since rEpsilon is soluble, the supernatant of the lysis buffer was collected and then purified by Ni^+2^-affinity chromatography using manual system (GE Healthcare). Eluted fractions were verified by SDS-PAGE 12% and Western blot using anti-6xHis monoclonal antibody (Sigma-Aldrich), and then mixed and dialyzed against phosphate buffered saline (PBS). Finally, the protein was quantified by BCA kit (Thermo Scientific), lyophilized and stored at 4 °C until use.

### Vaccine formulation and safety

To formulate the vaccines, lyophilized proteins were suspended in PBS and then mixed such that each 1.5 mL contained 200 μg of each of the three antigens. Aluminum hydroxide 2.5–3.5% (w/v) (Omega Chemicals) was used as adjuvant by diluting its suspension at a ratio of 1:2 with the antigens. The mixture was kept under slight agitation for 20 h at 25 °C for proper homogenization^23^. This way, each 3 mL contained one dose of the vaccine with 200 μg of each of the three recombinant antigens.

Sterility was verified by inoculating 0.5 mL of the vaccine formulation into four tubes containing 20 mL thioglycolate broth and four tubes containing 20 mL of Sabouraud broth. Two tubes with thioglycolate broth were incubated under anaerobic conditions. The remaining tubes were incubated aerobically. All tubes were kept at 37 °C for 21 days with daily OD_600_ readings^26^.

The innocuity of rAlpha and rBeta was determined *in vivo*, as previously described^9^. For rEpsilon, a cytotoxicity assay was performed *in vitro* using MDCK cell line, as previously described^27^. Briefly, MDCK cells were grown to confluence in 96-well culture plates (TPP) at 37 °C with 5% CO_2_ in DMEM low glucose with 10% fetal bovine serum (Sigma-Aldrich). Four different volumes of rEpsilon (50, 120, 210, and 375 ng/mL) were incubated with cells in a final volume of 100 μL for 1 h. Further, 50 μL of MTT solution (5 mg/mL in sterile PBS) were added and incubated for more than 3 h. The medium was removed, 200 μL of DMSO (dimethyl sulfoxide, Sigma-Aldrich) was added to each well, and optical densities were read at 540 nm. Positive controls for cell lysis were incubated with PBS + 0.1% Triton X-100 diluted 1:2 with medium, while negative controls received only PBS diluted 1:10 with medium.

### Potency test in rabbits

The potency test was performed using New Zealand female rabbits that each weighed approximately 1.5 Kg. Three groups of eight rabbits were tested as follows: Group 1 (G1, recombinant vaccine) received the formulation described in the “vaccine formulation and safety” section; Group 2 (G2, commercial vaccine) received a commercial vaccine (Covexin^®^ 9, Schering-Plough) for the purposes of comparing the results of Group 1 and Group 2; Group 3 (G3, negative control) received only sterile PBS mixed with Al(OH)_3_. The animals were subcutaneously immunized with a 10 × 15 needle on days zero and 21, and bleed on day 35 by cardiac puncture after xilazine-ketamine application (30 mg/Kg and 10 mg/Kg, respectively). The sera obtained from each group was pooled, resulting in one pooled serum per group. Each rabbit in Group 2 received the largest minimum dose of the commercial vaccine applied in the target species, in accordance with the vaccine testing legislation^11^,^12^,^26^. Since cattle received 3 mL of the commercial vaccine, while both sheep and goats received 2 mL, the inoculated amount in rabbits was 3 mL. According to information provided by the manufacturer, this vaccine also contains Al(OH)_3_ as adjuvant and can protect animals against nine different pathogens from *Clostridium* genus: *C. perfringens* types A, B, C, and D; *C. septicum*; *C. novyi*; *C. sordelli*; *C. chauvoei*; and *C. haemolyticum* D.

Pooled sera was used in the seroneutralization assay, which was performed by the Brazilian Agricultural Laboratory (LANAGRO), which is licensed for this test in accordance with the Brazilian Ministry of Agriculture, Livestock and Food Supply (MAPA) directive n° 23^24^, which regulates *C. perfringens* vaccine commerce in Brazil. The procedures were based on the United States Department of Agriculture (USDA)^10^, European Pharmacopoeia^11^, and Code of Federal Regulations Title 9 (CFR 9, USA)^12^ for measuring alpha, beta, and epsilon antitoxins, respectively. Briefly, 1 mL of each standard toxin (NIBSC) was incubated at 37 °C for 1 h with 1 mL of each pooled sera in serial dilutions from 1:1 to 1:32. Then, ten Swiss Webster mice weighing 18–22 g were intravenously inoculated with 0.2 mL of each sample and subsequently observed for 72 h for survival and then euthanized if necessary. The procedure was repeated with intermediary dilutions of the sera to identify the lower protective dilution. The survival information was used to calculate the IC_50_ and measure the results in international units per mL (IU/mL)^28^.

### Immunization of farm animals and quantification of neutralizing antibodies

All farm animals included in this study were 18-month-old males from the following species: Girolando cattle, Santa Inês sheep, and Toggenburg goats. Animals were fed by grazing in pastures with controlled minerals, and water was supplied freely. Groups were the same as those employed in the potency test in rabbits, but this time there were seven animals per group. The animals received the same recombinant vaccine formulation described in the “vaccine formulation and safety” section. The cattle in Group 2 received 3 mL of the commercial vaccine, while both sheep and goats received 2 mL. Each animal was immunized subcutaneously with a 10 × 15 needle on days zero and 28, and sera were collected four weeks after the last dose (on day 56). For the mice, a seroneutralization test was performed using each serum individually, instead of using a pool per group. The serum dilutions and incubation with native toxins, as well as injection in mice and calculation of IU/mL, were performed as per the process described for the potency test.

All animal experiments were performed in accordance with the guidelines of the National Council for Animal Experimentation Control (CONCEA) and the Ethics Committee in Animal Experimentation of the Federal University of Pelotas (CEEA-UFPel). In terms of the latter, the project was approved and completed under permit no. 7542.

### Statistical analysis

Neutralizing antibody levels generated by recombinant and commercial vaccines were compared using an unpaired, two-tailed, Mann-Whitney non-parametric test to determine significant differences in the immune response against each toxin each of the ruminant species exhibited. Moreover, the antibody levels generated in rabbits were compared with those generated in cattle, sheep, and goats using a paired, two-tailed, Wilcoxon non-parametric test to determine significant differences between each of the toxins for both commercial and recombinant vaccines. Since all animals of the group of goats immunized with commercial vaccine had the same antibody titers against alpha toxin, these values were the only exception to the statistics. For the first comparison, a paired, two-tailed, Wilcoxon non-parametric test was performed, while for the second, no test was applicable. The software Prism v5.01 (GraphPad) was used to calculate significant differences.

## Additional Information

**How to cite this article**: Moreira, G. M. S. G. *et al*. Immunogenicity of a Trivalent Recombinant Vaccine Against *Clostridium perfringens* Alpha, Beta, and Epsilon Toxins in Farm Ruminants. *Sci. Rep.* **6**, 22816; doi: 10.1038/srep22816 (2016).

## Supplementary Material

Supplementary Information

## Figures and Tables

**Table 1:** Anti-toxin levels detected on the potency test.

Vaccine	Anti-toxin level (IU/mL)		
	Alpha	Beta	Epsilon
Recombinant	9.6	24.4	25.0
Commercial	6.9	22.4	12.0

The minimum value against alpha, beta, and epsilon toxins are 4, 10, and 2 IU/mL, respectively, according to MAPA.

**Table 2:** Anti-toxin levels detected in cattle after immunization.

Vaccine	Anti-toxin level per animal (IU/mL)		
	Alpha	Beta	Epsilon
Recombinant	4.8	14.4	12.0
	5.7	14.4	14.4
	4.8	12.0	14.4
	4.8	14.4	14.4
	5.7	14.4	12.0
	5.7	14.4	12.0
	4.8	12.0	10.0
Mean ± SD	5.19 ± 0.48	13.71 ± 1.17	12.74 ± 1.70
Commercial	4.0	12.0	5.0
	< 4.0	10.0	6.0
	4.0	12.0	5.0
	< 4.0	< 10.0	6.0
	4.8	10.0	6.0
	4.0	14.4	7.2
	< 4.0	< 10.0	5.0
Mean ± SD	4.11 ± 0.30	11.20 ± 1.70	5.74 ± 0.81

The minimum value against alpha, beta, and epsilon toxins are 4, 10, and 2 IU/mL, respectively, according to MAPA. SD, standard deviation.

**Table 3:** Anti-toxin levels detected in sheep after immunization.

Vaccine	Anti-toxin level per animal (IU/mL)		
	Alpha	Beta	Epsilon
Recombinant	4.0	12.0	7.2
	4.0	14.4	10.0
	4.0	14.4	10.0
	4.0	14.4	7.2
	4.8	14.4	6.0
	4.8	12.0	6.0
	4.8	14.4	7.2
Mean ± SD	4.34 ± 0.43	13.71 ± 1.17	7.66 ± 1.69
Commercial	4.8	12.0	5.0
	4.8	12.0	5.0
	< 4.0	10.0	5.0
	< 4.0	< 10.0	5.0
	4.0	14.4	6.0
	4.0	10.0	5.0
	< 4.0	14.4	5.0
Mean ± SD	4.23 ± 0.39	11.83 ± 1.97	5.14 ± 0.38

The minimum value against alpha, beta, and epsilon toxins are 4, 10, and 2 IU/mL, respectively, according to MAPA. SD, standard deviation.

**Table 4:** Anti-toxin levels detected in goat after immunization.

Vaccine	Anti-toxin level per animal (IU/mL)		
	Alpha	Beta	Epsilon
Recombinant	4.8	14.4	10.0
	4.0	12.0	12.0
	4.8	14.4	10.0
	5.7	14.4	10.0
	4.0	12.0	7.2
	4.8	14.4	6.0
	4.8	14.4	7.2
Mean ± SD	4.70 ± 0.58	13.71 ± 1.17	8.91 ± 2.14
Commercial	4.0	< 10.0	5.0
	< 4.0	14.4	7.2
	4.0	10.0	6.0
	4.0	14.4	6.0
	< 4.0	14.4	6.0
	4.0	10.0	5.0
	4.0	12.0	7.2
Mean ± SD	4.00 ± 0.00	12.17 ± 2.20	6.06 ± 0.90

The minimum value against alpha, beta, and epsilon toxins are 4, 10, and 2 IU/mL, respectively, according to MAPA. SD, standard deviation.
