# Supplementary material for: Immunogenicity of a Trivalent Recombinant Vaccine Against Clostridium perfringens Alpha, Beta, and Epsilon Toxins in Farm Ruminants
Source: Sci Rep. 2016 Mar 23;6:22816. doi: 10.1038/srep22816 (PMC4804304; doi:10.1038/srep22816)
Supplement: Supplementary Information [file srep22816-s1.pdf]

Supplementary Information

Immunogenicity of a Trivalent Recombinant Vaccine Against *Clostridium perfringens* Alpha, Beta, and Epsilon Toxins in Farm Ruminants

Gustavo Marçal Schmidt Garcia Moreira[1,5,+], Felipe Masiero Salvarani[2,+], Carlos Eduardo Pouey da Cunha[1], Marcelo Mendonça[1], Ângela Nunes Moreira[3], Luciana Aramuni Gonçalves[4], Prhiscylla Sadanã Pires[4], Francisco Carlos Faria Lobato[4], Fabricio Rochedo Conceição[1,*]

[1]Centro de Desenvolvimento Tecnológico, Universidade Federal de Pelotas, Pelotas, Rio Grande do Sul, CEP 96160-000, Brazil
[2]Instituto de Medicina Veterinária, Universidade Federal do Pará, Castanhal, Pará, CEP 68740-970, Brazil
[3]Faculdade de Nutrição, Universidade Federal de Pelotas, Pelotas, Rio Grande do Sul, CEP 96010-610, Brazil
[4]Escola de Veterinária, Universidade Federal de Minas Gerais, Belo Horizonte, Minas Gerais, CEP 30123-970, Brazil
[5]Current address: Technische Universität Braunschweig, Institut für Biochemie, Biotechnologie und Bioinformatik, Abteilung Biotechnologie, Spielmannstr. 7, 38106 Braunschweig, Germany
*fabricio.rochedo@ufpel.edu.br
+these authors contributed equally to this work

Figures

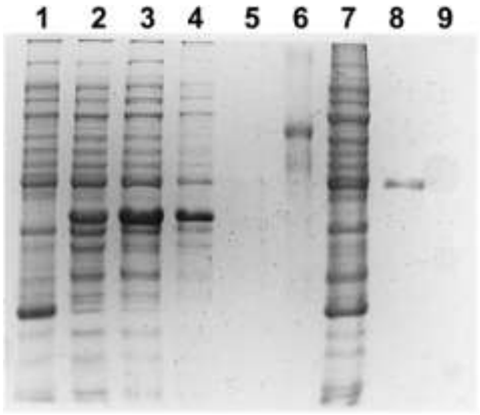

Figure S1. Complete figure of the SDS-PAGE 12 % containing rEpsilon expression in *E. coli*. 1- *E. coli* BL21 (DE3) Star not transformed; 2- induced *E. coli* BL21 (DE3) Star transformed with pAE-rEpsilon; 3- supernatant of lysis buffer of induced *E. coli* BL21 (DE3) Star containing pAE-rEpsilon; 4- pellet fraction of induced *E. coli* BL21 (DE3) Star containing pAE-rEpsilon after lysis; 5- empty well; 6- BSA (67 kDa); 7- *E. coli* expressing a 42-kDa protein; 8- purified recombinant 42-kDa protein; 9- empty well. Of note, only wells 1-4 and 6 were included in Fig. 1A of the article.

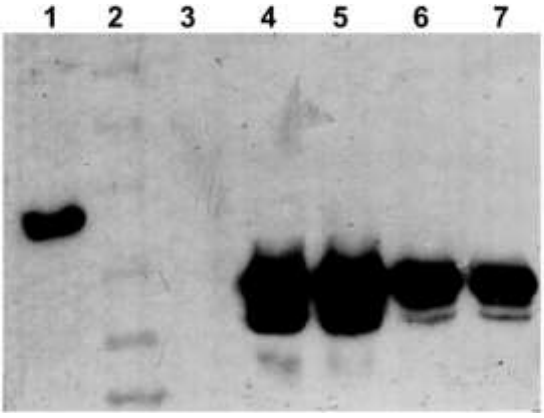

Figure S2. Complete figure of the Western blot using anti-6xHis antibody to detect purified rEpsilon. 1- purified recombinant 50-kDa protein; 2- PageRuler Prestained Protein Ladder (Thermo Scientific); 3-7- different elution fractions purified from supernatant of lysis buffer of induced *E. coli* BL21 (DE3) Star containing pAE-rEpsilon. Of note, only wells 2 and 7 were included in Fig. 1C of the article.
